# Isolation, Sequence, Infectivity, and Replication Kinetics of Severe Acute Respiratory Syndrome Coronavirus 2

**DOI:** 10.3201/eid2609.201495

**Published:** 2020-09

**Authors:** Arinjay Banerjee, Jalees A. Nasir, Patrick Budylowski, Lily Yip, Patryk Aftanas, Natasha Christie, Ayoob Ghalami, Kaushal Baid, Amogelang R. Raphenya, Jeremy A. Hirota, Matthew S. Miller, Allison J. McGeer, Mario Ostrowski, Robert A. Kozak, Andrew G. McArthur, Karen Mossman, Samira Mubareka

**Affiliations:** McMaster University, Hamilton, Ontario, Canada (A. Banerjee, J.A. Nasir, K. Baid, A.R. Raphenya, J.A. Hirota, M.S. Miller, A.G. McArthur, K. Mossman);; University of Toronto, Toronto, Ontario, Canada (P. Budylowski, N. Christie, A. Ghalami, A.J. McGeer, M. Ostrowski, R.A. Kozak, S. Mubareka);; Sunnybrook Research Institute, Toronto (L. Yip, P. Aftanas, R.A. Kozak, S. Mubareka);; Mount Sinai Hospital, Toronto (A.J. McGeer)

**Keywords:** 2019 novel coronavirus disease, coronavirus disease, COVID-19, severe acute respiratory syndrome coronavirus 2, SARS-CoV-2, viruses, respiratory infections, zoonoses, isolation, replication, immune cells, phylogenetics

## Abstract

Since its emergence in Wuhan, China, in December 2019, severe acute respiratory syndrome coronavirus 2 (SARS-CoV-2) has infected ≈6 million persons worldwide. As SARS-CoV-2 spreads across the planet, we explored the range of human cells that can be infected by this virus. We isolated SARS-CoV-2 from 2 infected patients in Toronto, Canada; determined the genomic sequences; and identified single-nucleotide changes in representative populations of our virus stocks. We also tested a wide range of human immune cells for productive infection with SARS-CoV-2. We confirm that human primary peripheral blood mononuclear cells are not permissive for SARS-CoV-2. As SARS-CoV-2 continues to spread globally, it is essential to monitor single-nucleotide polymorphisms in the virus and to continue to isolate circulating viruses to determine viral genotype and phenotype by using in vitro and in vivo infection models.

Severe acute respiratory syndrome coronavirus 2 (SARS-CoV-2) emerged in December 2019 in Wuhan, China (*1*). SARS-CoV-2 has since spread to ≈185 countries and infected ≈6 million persons, among whom ≈380,000 have died (*2*). On January 23, 2020, a case of coronavirus disease (COVID-19) was detected in Toronto, Canada (*3*); since then, multiple cases have been identified across Canada. As SARS-CoV-2 spreads globally, the virus is likely to adapt and evolve. It is critical to isolate SARS-CoV-2 viruses to characterize their ability to infect and replicate in multiple human cell types and to determine if the virus is evolving in its ability to infect human cells and cause severe disease. Isolating the virus also provides the opportunity to share the virus with other researchers for development and testing of diagnostics, drugs, and vaccines.

We isolated SARS-CoV-2 from 2 patients with COVID-19 and determined the genomic sequence of each isolate (SARS-CoV-2/SB2 and SARS-CoV-2/SB3-TYAGNC). In addition, we studied the replication kinetics of SARS-CoV-2/SB3-TYAGNC in human fibroblast, epithelial, and immune cells. 

## Methods

### Cells

We maintained Vero E6 cells (African green monkey cells; American Type Culture Collection [ATCC], https://www.atcc.org) in Dulbecco’s modified Eagle medium (DMEM) supplemented with 10% fetal bovine serum (FBS) (Sigma-Aldrich, https://www.sigmaaldrich.com) and 1× l-glutamine and penicillin/streptomycin (Pen/Strep; Corning, https://ca.vwr.com). Calu-3 cells (human lung adenocarcinoma derived; ATCC) were cultured as previously mentioned (*4*), as were THF cells (human telomerase life-extended cells) (*5*). THP-1 cells (monocytes; ATCC) were cultured in RPMI medium (Gibco Laboratories, https://www.thermofisher.com) supplemented with 10% FBS, 2mM l-glutamine, 1× penicillin/streptomycin, and 0.05 mM β-mercaptoethanol. THP-1 cells (monocytes and differentiated macrophages and dendritic cells) were differentiated into macrophages by using 50 ng/mL lymphocyte/granulocyte/macrophage–colony stimulating factor (LGM-CSF; R&D Systems, https://www.rndsystems.com) plus 50 ng/mL macrophage–colony stimulating factor (R&D Systems) and into dendritic cells by using 50 ng/mL granulocyte/macrophage-colony stimulating factor (GM-CSF; R&D Systems) plus 500 U/mL interleukin-4 (BioLegend, https://www.biolegend.com). We purified peripheral blood mononuclear cells (PBMCs) from 2 healthy donors (OM8066 and OM8067) into CD4+, CD8+, CD19+, monocytes, and other cells (CD4*–,* CD8–, CD19–) by using a CD4+ selection kit that uses immunomagnetic negative selection, a CD8+ selection kit, a phycoerythrin-positive selection kit, and a monocyte-negative selection kit, all by STEMCELL Technologies (https://www.stemcell.com; Appendix Figure 1). We resuspended CD4+, CD8+, CD19+ and CD4–, CD8–, CD19– cells in R-10 media (RPMI + 2 mM l-glutamine + 10% FBS + penicillin/streptomycin) plus 20 U/mL interleukin-2. Primary monocytes were resuspended in R-10 media. This work was approved by the Sunnybrook Research Institute Research Ethics Board (149–1994) and the Research Ethics Boards of St. Michael’s Hospital and the University of Toronto (REB 20–044; for PBMCs).

### Isolation and Quantification

We seeded Vero E6 cells at a concentration of 3 × 10^5^ cells/well in a 6-well plate. The next day, we collected 200 μL of mid-turbinate swab samples from 2 COVID-19 patients, mixed it with 200 μL of DMEM containing 16 μg/mL TPCK-treated trypsin and inoculated the cells. After 1 h, the inoculum was replaced with DMEM containing 2% FBS and 6 μg/mL TPCK-treated trypsin. We observed the cells daily under a light microscope. Supernatant from the cells was used to determine virus titers (50% tissue culture infectious dose [TCID_50_]/mL) according to the Spearman and Karber method (*6*,*7*) as outlined previously (*8*).

### Quantitative Real-Time PCR

To detect SARS-CoV-2 in cell culture supernatant, we removed 140 μL of supernatant and performed detection of viral nucleic acids by reverse transcription PCR (RT-PCR), following an adaptation of the Corman et al. protocol (*9*). In brief, we extracted viral RNA from infected cells by using a QIAamp viral RNA kit (QIAGEN, https://www.qiagen.com) according to the manufacturer’s instructions. The RT-PCR reactions were conducted by using Luna Universal qPCR Master Mix (New England Biolabs, https://www.neb.ca) according to the manufacturer’s instructions. Two separate gene targets were used for detection, the 5′ untranslated region (UTR) and the envelope (E) gene. Primers and probes used were 5′ UTR forward GTTGCAGCCGATCATCAGC, 5′ UTR reverse GACAAGGCTCTCCATCTTACC, and 5′ UTR probe FAM-CGGTCACACCCGGACGAAACCTAG-BHQ-1; and E-gene forward CAGGTACGTTAATAGTTAATAGCGT, E-gene reverse ATATTGCAGCAGTACGCACACA, and E-gene probe CAL Fluor Orange 560-ACACTAGCCATCCTTACTGCGCTTCG-BHQ-1. The cycling conditions were 1 cycle of denaturation at 60°C for 10 min, then 95°C for 2 min, followed by 44 amplification cycles at 95°C for 10 s and 60°C for 15 s. Analysis was performed by using Rotor-Gene Q software (QIAGEN) to determine cycle threshold (C_t_).

### Electron Microscopy

Samples were fixed in 10% neutral buffered formalin (Sigma-Aldrich), for 1 h. Pellets were washed with 0.1 M phosphate buffer (pH 7.0) and postfixed with 1% osmium tetroxide in 0.1 M phosphate buffer (pH 7.0) for 1 h. Pellets were washed with distilled water and en-bloc stained with 2% uranyl acetate in distilled water for 2 h. Pellets were washed with distilled water and dehydrated in a series of ethanol concentrations**.** Pellets were infiltrated with Araldite Embed 812 resin (VWR, https://us.vwr.com) and cured at 65°C for 48 h. Resin blocks were trimmed, polished, and 9 nm thin sections were ultramicrotomed (Leica Reichert Ultracut E, https://www.leica-microsystems.com) and mounted on transmission electron microscopy grids. Thin sections were stained with 5% uranyl acetate and 5% lead citrate. Sections were imaged by using transmission electron microscopy (Talos L120C; ThermoFisher Scientific, https://www.thermofisher.com) and an LaB6 (lanthanum hexaboride) filament at 120 kV. We scanned 10 fields per cell type, each at a different magnification level: 2,600×, 8,500×, 17,500×, and 36,000×.

### Immunofluorescence

**Figure 1:**
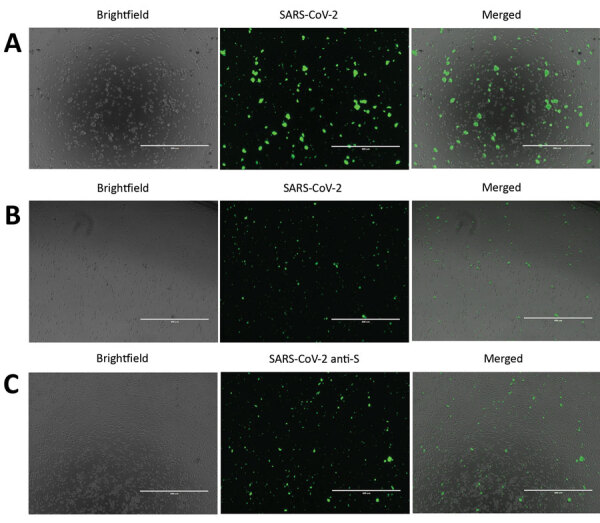
Severe acute respiratory syndrome coronavirus 2 (SARS-CoV-2) protein detection in infected Vero E6 and CD4+ T cells. To detect SARS-CoV-2 protein expression, we infected Vero E6 and CD4+ T cells with SARS-CoV-2 at a multiplicity of infection of 0.1 for 24 h. We immunostained these cells and observed them by using fluorescent microscopy. A) SARS-CoV-2–infected and immunostained Vero E6 cells. B) SARS-CoV-2–infected and immunostained CD4+ T cells. For panels A and B, cells were stained by using an antibody cocktail consisting of SARS-CoV-2 S1 antibody, SARS-CoV-2 N antibody, and diluted serum from a recovered coronavirus disease patient. C) SARS-CoV-2 infected CD4+ T cells immunostained with SARS-CoV-2 S1 antibody (anti-S). Scale bars indicate 400 μm; original magnification ×10.

To detect SARS-CoV-2 proteins in Vero E6 and CD4+ T lymphocytes, we infected cells with SARS-CoV-2 at a 0.1 multiplicity of infection (MOI) for 24 h. After 24 h, we fixed the cells in 10% neutral buffered formalin (Sigma-Aldrich). After fixation, cells were permeabilized and blocked as previously described (*10*). Cells were stained in suspension by using a previously described protocol (*10*). For primary antibody staining, we used a combination of 6.6 μg/mL rabbit anti-SARS-CoV-2 N (BioVision, https://www.biovision.com) plus 10 μg/mL recombinant human anti-SARS-CoV-2 spike S1 (GenScript, https://www.genscript.com) and 1:100 diluted serum from a recovered COVID-19 patient (OM8073) (Figure 1, panels A, B). To confirm SARS-CoV-2 staining in CD4+ T cells, we used 10 μg/mL recombinant human SARS-CoV-2 spike S1 antibody as primary staining antibody (GenScript) alone (Figure 1, panel C). For secondary antibodies, we used 1 μg/mL mouse anti-human FITC (BioLegend) and 4 μg/mL goat anti-rabbit Alexa Fluor 488 (abcam, https://www.abcam.com). After staining, cells were spun at 500 × *g* for 5 min in a 96-well plate. The cells were observed under an EVOS FL digital microscope (VWR).

### Flow Cytometry

To prepare cells for flow cytometry, we washed 100 μL (400,000 cells) of primary CD4+, CD8+, and CD19+ cells and monocytes with 1 mL of phosphate-buffered saline (PBS) and spun the cells at 500 *g* for 5 min. The cells were resuspended in 100 μL of Live/Dead Violet stain (ThermoFisher Scientific) according to the manufacturer’s recommendation and diluted 1:1,000 in PBS. Cells were incubated at 4°C for 30 min. Next, cells were washed with 1 mL of fluorescence-activated cell sorting buffer (in-house reagent) and spun at 500 × *g* for 5 min. Cells were then stained with 100 μL of their respective stains (αCD4-FITC, αCD8-FITC, αCD19-FITC, αCD14-APC; BioLegend) at a concentration of 1 μg/mL for 30 min at 4°C. After staining, the cells were washed with 1 mL of fluorescence-activated cell sorting buffer and spun at 500 × *g* for 5 min. Extra aliquots of cells were left unstained and also spun at 500 × *g* for 5 min. The pellets were resuspended in 100 μL of 1% paraformaldehyde (ThermoFisher Scientific) and analyzed. Samples were run on the BD LSRFortessa X-20 (BD, https://www.bdbiosciences.com). To exclude debris and dead cells, we stained the cells with Live/Dead Violet stain, which stains dead cells brightly. Cells were then analyzed on a flow cytometer, and brightly stained cells were excluded. The remaining cells were then analyzed for the expression of their respective cell surface markers to assess purity. 

### Sequencing and Phylogenetic Relationship

RNA was extracted from the supernatant of Vero E6 cells after 1 passage by using the QIAamp Viral RNA Mini kit (QIAGEN) without addition of carrier RNA. We synthesized double-stranded DNA for sequencing library preparation by using the Liverpool SARS-CoV-2 amplification protocol (*11*). Two 100-μM primer pools were prepared by combining primer pairs in an alternating fashion to prevent amplification of overlapping regions in a single reaction. In a PCR tube, we added 1 μL Random Primer Mix (ProtoScript II First Strand cDNA Synthesis Kit; New England Biolabs) to 7 μL extracted RNA and denatured it on a SimpliAmp Thermal Cycler (ThermoFisher Scientific) at 65°C for 5 min and then incubated it on ice. We then added 10 μL 2X ProtoScript II Reaction Mix and 2 μL 10X ProtoScript II Enzyme Mix to the denatured sample and performed cDNA synthesis under the following conditions: 25°C for 5 min, 48°C for 15 min, and 80°C for 5 min. After cDNA synthesis, in a new PCR tube we combined 2.5 µL cDNA with 12.5 µL Q5 High-Fidelity 2X Master Mix (New England Biolabs), 8.8 µL nuclease-free water (ThermoFisher Scientific), and 1.125 µL of 100 µM primer pool 1 or 2. PCR cycling was then performed as follows: 98°C for 30 s, followed by 40 cycles of 98°C for 15 s and 65°C for 5 min.

All PCRs were purified by using RNAClean XP (Beckman Coulter, https://www.beckmancoulter.com) at a 1.8× bead-to-amplicon ratio and eluted in 30 μL of RNase-free water (AmericanBio, https://www.americanbio.com). We quantified 2 μL of amplified material by using a Qubit 1X dsDNA assay (ThermoFisher Scientific) according to the manufacturer’s instructions. Illumina sequencing libraries were prepared by using a Nextera DNA Flex Library Prep Kit and Nextera DNA CD Indexes (Illumina, https://www.illumina.com) according to the manufacturer’s instructions. Paired-end 150-bp sequencing was performed for each library on a MiniSeq with a 300-cycle mid-output reagent kit (Illumina), multiplexed with targeted sampling of ≈40,000 clusters per library. Sequencing reads from pools 1 and 2 were combined (as R1 and R2), amplification primer sequences were removed by using Cutadapt version 1.18 (*12*), and Illumina adaptor sequences were removed and low-quality sequences trimmed or removed by using Trimmomatic (version 0.36) (*13*). Final sequence quality and confirmation of adaptor/primer trimming were confirmed by using FASTQC version 0.11.5 (*14*). SARS-CoV-2 genome sequences were assembled by using UniCycler version 0.4.8 (default settings, except for conservative mode) (*15*) and assembly statistics were generated by QUAST (version 5.0.2) (*16*). Sequencing depth and completeness of coverage of the assembled genomes was additionally assessed by using Bowtie2 version 2.3.4.1 (*17*) alignment of the sequencing reads against the assembled contigs, and statistics were generated by ngsCAT (version 0.1) (*18*). Sequence variation in the assembled genomes was assessed by comparing sequences in BLASTN (http://blast.ncbi.nlm.nih.gov//Blast.cgi) with SARS-CoV-2 genomes available in GenBank as well as BreSeq version 0.35.0 (*19*) analysis relative to GenBank entry MN908947.3 (first genome sequence reported from the original outbreak in Wuhan). We constructed a phylogenetic tree (Appendix Figure 2) by using maximum-likelihood based on a multiple sequence alignment and RAxML-HPC BlackBox with the general time-reversible plus gamma plus invariate sites model for among-site rate variation (*20*).

## Results

**Figure 2:**
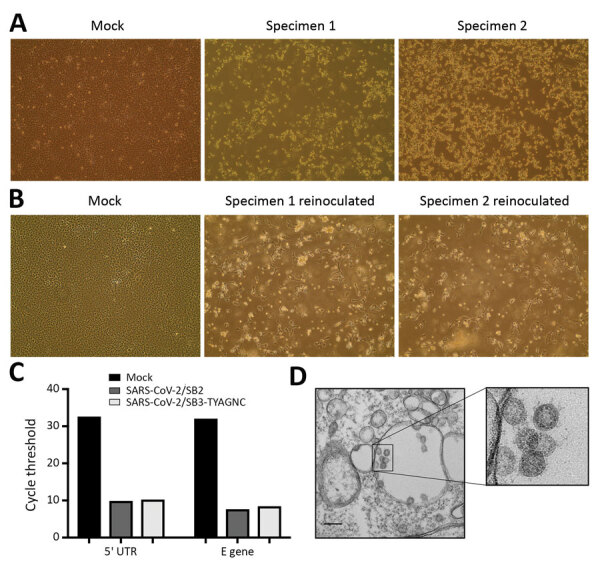
Isolating severe acute respiratory syndrome coronavirus 2 (SARS-CoV-2) from patients with coronavirus disease (COVID-19). A) Vero E6 cells were mock inoculated or inoculated with midturbinate clinical specimens from COVID-19 patients. Cells were incubated for 72 h and observed for cytopathic effect (CPE) under a light microscope. Original magnification ×10. B) To determine if supernatant from Vero E6 cells that were mock inoculated or inoculated with clinical specimens contained replication competent virus, we reinoculated a fresh monolayer of Vero E6 cells and observed cells under a light microscope for CPE after 24 h. Original magnification ×10. C) Quantitative real-time PCR was used to detect SARS-CoV-2 5′-UTR and E gene in RNA extracted from supernatant that was collected from Vero E6 cells that were mock infected or infected with clinical specimens from COVID-19 patients for 72 h. D) Electron micrograph of Vero E6 cells that were reinfected for 48 h with supernatant that was collected from Vero E6 cells infected with clinical specimens. Original magnification ×36,000. Inset, zoomed and cropped from the original electron micrograph, shows coronavirus-like particles. M, mock specimen; specimen 1, SARS-CoV-2/SB2; specimen 2, SARS-CoV-2/SB3-TYAGNC. E, envelope; UTR, untranslated region.

For virus isolation, we inoculated Vero E6 cells with aliquots of mid-turbinate swab samples and monitored the cells for cytopathic effects (CPE) daily. Relative to mock-inoculated cells, cells inoculated with both samples (SARS-CoV-2/SB2 and SARS-CoV-2/SB3-TYAGNC) displayed extensive CPE 72 h after infection (Figure 2, panel A). We collected 200 μL of cell culture supernatant and re-infected a fresh layer of Vero E6 cells. After 24 hours, both wells containing cells that were reinoculated displayed extensive CPE (Figure 2, panel B). We extracted viral RNA from the supernatant and confirmed the presence of SARS-CoV-2 by using a diagnostic quantitative real-time PCR (Figure 2, panel C). We also confirmed the presence of coronavirus-like particles in infected Vero E6 cells by electron microscopy (Figure 2, panel D).

Next, we performed genome sequencing of both isolates, generating genome sequences with 7,500-8,000–fold coverage and ≈94% completeness, with only ≈260 bp and ≈200 bp at the 5′ and 3′ termini undetermined (Table; Appendix Figure 2). SARS-CoV-2/SB2 and SARS-CoV-2/SB3-TYAGNC shared synonymous and nonsynonymous substitutions with those independently observed in direct sequencing of clinical isolates (Table; S. Mubareka and A.G. McArthur, unpub. data). SARS-CoV-2/SB2 also contained a nonsynonymous substitution at position 2832 (K856R in open reading frame [ORF] 1ab polyprotein) and 3 regions with mutations or a deletion supported by a minority of sequencing reads, but SARS-CoV-2/SB3-TYAGNC had only an additional synonymous substitution in ORF1ab polyprotein (Y925Y) plus a minority of sequencing reads supporting another synonymous substitution in the ORF3a protein (Table). Furthermore, maximum-likelihood phylogenetic analysis including >1,900 SARS-CoV-2 isolates from GISAID (https://www.gisaid.org) placed both SARS-CoV-2/SB2 and SARS-CoV-2/SB3-TYAGNC within a clade of isolates from patients around the globe but with evidence of travel history associated with the COVID-19 outbreak in Iran (Appendix Figure 2). As such, SARS-CoV-2/SB3-TYAGNC was used for subsequent studies as the best representative of a clinical viral isolate. Raw sequencing reads for each isolate are available in the National Center for Biotechnology Information under BioProject PRJNA624792. Only sequencing reads that aligned by Bowtie2 to the MN908947.3 SARS-CoV-2 genome were included in the deposited sequence files.

**Table:** Sequencing read and genome assembly statistics used in study of isolation, sequence, infectivity, and replication kinetics of SARS-CoV-2*

Metric or mutation	SARS-CoV-2/SB2	SARS-CoV-2/SB3_TYAGNC
Number of paired reads	730,137 bp	690,167 bp
Reads from SARS-CoV-2	94.0%	94.4%
Number of assembly contigs	1	1
Assembly N50	29,494 bp	29,369 bp
Average depth of coverage of reads	7940.0-fold	7550.1-fold
Total assembly length	29,494 bp	29,369 bp
SARS-CoV-2 assembly completeness	98.6%	98.2%
Unresolved 5′ sequence	262 bp	272 bp
Unresolved 3′ sequence	200 bp	205 bp
Pos. 884 (orf1ab polyprotein)		**R207C (CGT→TGT)**
Pos. 1397 (orf1ab polyprotein)	**V378I (GTA→ATA)**	**V378I (GTA→ATA)**
Pos. 2832 (orf1ab polyprotein)	K856R (AAG→AGG)	
Pos. 3040 (orf1ab polyprotein)		Y925Y (TAC→TAT)
Pos. 8327 (or1ab polyprotein)	18.1% of reads suggest L2688F (CTT→TTT)	
Pos. 8653 (orf1ab polyprotein)		**M2796I (ATG→ATT)**
Pos. 10353 (orf1ab polyprotein)	5.6% of reads suggest K3363T (AAG→ACG)	
Pos. 11074 (orf1ab polyprotein)	10.2% of reads suggest +TTT and a deletion between positions 10809 and 13203	
Pos. 11083 (orf1ab polyprotein)	**L3606F (TTG→TTT)**	**L3606F (TTG→TTT)**
Pos. 25413 (orf3a protein)		36.7% of reads suggest I7I (ATC→ATT)
Pos. 28688 (nucleocapsid phosphoprotein)	**L139L (TTG→CTG)**	**L139L (TTG→CTG)**

*Predicted mutations are relative to the MN908947.3 SARS-CoV-2 genome (29,903 bp). Mutations within codons are underlined. All mutations were predicted by 100% of sequencing reads mapping to that position, unless otherwise noted. None of the mutations with support from <100% of sequencing reads appeared in the final assembled genome consensus sequences. Substitutions in boldface have been observed in direct sequencing of patient isolates (S. Mubareka, A.G.McArthur, unpub. data). orf1ab, open reading frame 1ab; pos., position; SARS-CoV-2, severe acute respiratory syndrome coronavirus 2.

**Figure 3:**
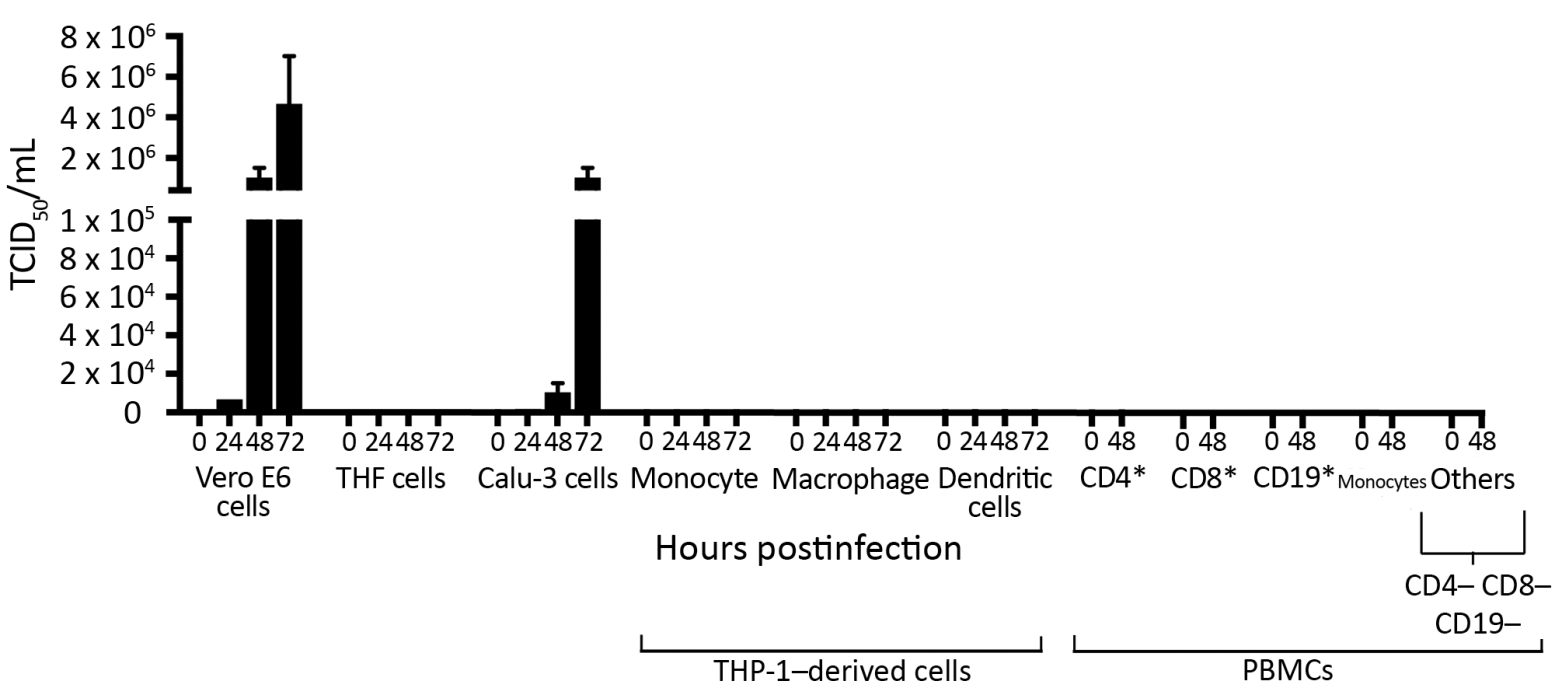
Replication of severe acute respiratory syndrome coronavirus 2 (SARS-CoV-2) in human structural and immune cells. To identify human cells that support SARS-CoV-2 replication, we infected human cell lines and primary cells at a multiplicity of infection of 0.01 (n = 2 independent experiments; supernatant from each experiment was titrated in triplicate). We infected Vero E6 cells as a control. THF (human telomerase life-extended cells) and Calu-3 cells (human lung adenocarcinoma–derived) cells represent human structural cells. THP-1 is a monocyte cell line that was used to derive macrophages and dendritic cells. PBMCs from 2 healthy human donors were used to generate CD4+, CD8+, CD19+, monocytes, and other (CD4–, CD8–, CD19–) cell populations. Supernatant from infected cells was collected at various times and titrated on Vero E6 cells to determine virus titers (TCID_50_). PBMC, peripheral blood mononuclear cell; TCID_50_, 50% tissue culture infectious dose.

To determine the replication kinetics of SARS-CoV-2 in human structural and immune cells, we infected Calu-3 cells, THF cells, Vero E6 cells (African green monkey kidney epithelial), THP-1 cells, and primary PBMCs from healthy human donors (CD4+, CD8+, CD19+, monocytes, and other PBMCs; Appendix Figure 1) with an MOI of 0.01. We monitored virus replication in the cell lines for 72 h (Figure 3). We also determined virus replication in PBMCs from healthy donors for 48 h (Figure 3). SARS-CoV-2 propagated to high titers in Vero E6 and Calu-3 cells (Figure 3). SARS-CoV-2 did not replicate efficiently in THF cells (Figure 3). Of note, human immune cell lines and primary PBMCs from healthy donors did not support SARS-CoV-2 replication (Figure 3).

**Figure 4:**
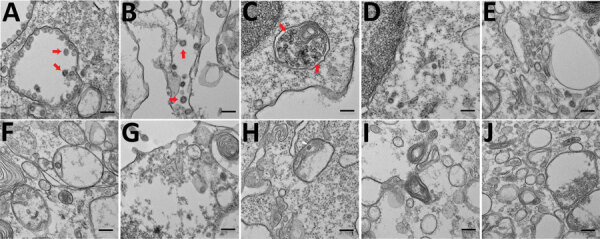
Electron micrographs of severe acute respiratory syndrome coronavirus 2 (SARS-CoV-2)–infected cells. To detect coronavirus-like particles in experimentally infected human structural and immune cells, we infected a range of cells with SARS-CoV-2 at a multiplicity of infection of 0.01 for 48 h. The cells were fixed, processed, and imaged by using a transmission electron microscope (10 fields/cell type). A representative image of each cell type is shown. Virus-like particles are indicated by red arrows. A) Vero E6 cells. B) Calu-3 cells. C) CD4+ PBMCs. D) CD8+ PBMCs. E) CD19+ PBMCs. F) Monocytes from PBMCs. G) Other cells from PBMCs (CD4–, CD8–, CD19– cell populations). H) THP-1 monocyte. I) THP-1-derived macrophage. J) THP-1-derived dendritic cell. PBMC, peripheral blood mononuclear cell. Scale bars indicate 200 nm.

To further support virus replication data, we imaged infected human epithelial, fibroblast, and immune cells by using electron microscopy after 48 h of infection with SARS-CoV-2 at an MOI of 0.01 (Figure 4). We scanned 10 different fields per cell type, each using 4 different magnifications—2,600×, 8,500×, 17,500×, and 36,000×—to determine if the cell populations contained virus-like particles. Virus-like particles were detected in 7/10 fields in Vero E6 cells and 8/10 fields in Calu-3 cells (Figure 4, panels A, B). We also detected virus-like particles in 2/10 fields in primary CD4^+^ T cells (Figure 4, panel C). We did not observe any virus-like particles in other human immune cells that were experimentally infected with SARS-CoV-2 (Figure 4, panels D–J). To determine if virus-like particles can be detected in Vero E6 cells and PBMCs at earlier time points, we infected these cell populations with SARS-CoV-2 at an MOI of 0.01 and imaged the cells with electron microscopy at 6 h and 12 h after infection (Appendix Figures 3, 4). We observed virus-like particles in 9/10 fields at 6 h after infection and 10/10 fields at 12 h after infection in Vero E6 cells (Appendix Figure 3, panel A, Figure 4, panel A). We also observed virus-like particles in 1/10 fields at 6 h and 1/10 fields at 12 h after infection in CD4+ T cells (Appendix Figure 3, panel B, and Figure 4, panel B). None of the other infected PBMC populations contained detectable virus-like particles (Appendix Figure 3, panels C–F, and Figure 4, panels C–F).

To confirm SARS-CoV-2 infection and protein expression in CD4+ T cells, we infected Vero E6 and CD4+ T cells with SARS-CoV-2 at an MOI of 0.1 for 24 h. We immunostained these cells and observed them by using fluorescent microscopy. To enhance our ability to detect SARS-CoV-2 proteins in these cells, we immunostained the cells by using a cocktail of antibodies that included SARS-CoV-2 S1 antibody, SARS-CoV-2 N antibody, and diluted serum from a recovered COVID-19 patient (Figure 1, panels A and B). We were able to detect SARS-CoV-2 infected Vero E6 and CD4+ T cells by using this antibody cocktail (Figure 1, panels A, B). Furthermore, to confirm SARS-CoV-2 infection of CD4+ T cells by using a single antibody, we immunostained infected CD4+ T cells with anti-SARS-CoV-2 S1 antibody and were able to detect infected cells in the population (Figure 1, panel C).

## Discussion

We report the isolation of 2 replication competent SARS-CoV-2 virus samples from COVID-19 patients in Canada. We used TPCK-treated trypsin to facilitate virus isolation from clinical specimens (Figure 2, panel A). Exogenous trypsin activates SARS-CoV spike proteins more efficiently and facilitates cellular entry (*21*). Exogenous trypsin treatment also enhances infectivity of other zoonotic batborne coronaviruses (*22*). Furthermore, TPCK-treated trypsin has been used to successfully isolate SARS-CoV-2 in China (*1*). In our study, subsequent infection and virus replication did not require any additional TPCK-treated trypsin (Figure 2, panel B). The presence of CPE alone does not indicate successful isolation of a coronavirus. Mid-turbinate samples from adults with acute respiratory distress may often contain other microbes, including viruses (*23*). Thus, to identify our cell culture isolates, we sequenced them to confirm that they were reflective of the SARS-CoV-2 infecting patients worldwide, selecting SARS-CoV-2/SB3-TYAGNC for experimental investigation because this isolate produced fewer minority sequencing reads (Table).

SARS-CoV caused the 2003–2004 outbreak of severe acute respiratory syndrome. SARS-CoV can infect structural (*24*) and immune cell lines (*25*) from humans in vitro. To identify cell types that can support productive infection of SARS-CoV-2, we infected a range of human cell populations with SARS-CoV-2/SB3-TYAGNC. Both Vero E6 and Calu-3 cells supported SARS-CoV-2 replication to high titers (Figure 3), as reported in other recent studies (*26*,*27*). Previously, SARS-CoV was also shown to replicate efficiently in Vero E6 cells (*24*). Vero E6 cells are immunodeficient, with deficiencies in innate antiviral interferon signaling, which makes them ideal candidates for virus isolation (*28*). However, to enable studies on SARS-CoV-2–host interactions, it is important to identify human lung epithelial cells with intact immune responses that can support SARS-CoV-2 replication. We and others have previously shown that SARS-CoV and Middle East respiratory syndrome coronavirus (MERS-CoV) replicate efficiently in Calu-3 cells (*8*,*29*,*30*). In addition, SARS-CoV–induced and MERS-CoV–induced immune responses have been studied in Calu-3 cells (*30*,*31*). The ability to infect Calu-3 cells with SARS-CoV-2 (Figure 3) will facilitate in vitro studies of virus–host interactions using SARS-CoV-2. Other commonly used human lung cells, such as A549, do not support efficient replication of SARS-CoV-2 (*26*). Furthermore, hTERT (human telomerase reverse transcriptase) THF cells also did not support virus replication (Figure 3).

Previous studies have shown that human immune cells, such as THP-1 cells, are susceptible to SARS-CoV infection (*25*). In our study, human immune cell populations, including THP-1–derived cell lines and primary cells (PBMCs) did not support productive SARS-CoV-2 replication (Figure 3). Although primary CD4+ T cells did not support productive virus replication, we observed virus-like particles in these cells by electron microscopy (Figure 4, panel C). We also detected SARS-CoV-2 proteins in infected CD4+ T cells by using fluorescent microscopy (Figure 1, panels B, C). This finding is consistent with that recently reported by Wang et al. when they demonstrated that SARS-CoV-2 and pseudotyped viruses could enter human T-cell lines (MT-2) (*32*). Those authors also noted that SARS-CoV-2 replication was abortive in MT-2 cells. SARS-CoV-2 transcript levels in infected MT-2 cells increased at 6 h after infection but remained steady at later time points, indicating a lack of virus replication in these cells (*32*). This finding is similar to abortive replication observed in MERS-CoV–infected T lymphocytes (*33*). However, the study by Wang et al. did not quantify virus titers in the supernatant from infected cells. In our study, we could not detect any replication-competent virus in the supernatant that was collected from SARS-CoV-2–infected CD4+ T cells (Figure 3). Human immune cells lack expression of angiotensin-converting enzyme 2 (*34*) (https://www.proteinatlas.org), the functional receptor of SARS-CoV-2 (*1*,*35*). Emerging data indicate that there could be other receptors, such as CD147, that may facilitate cellular entry of SARS-CoV-2 (K. Wang et al., unpub. data, https://www.biorxiv.org/content/10.1101/2020.03.14.988345v1). Additional studies are needed to determine the full breadth of cellular receptors and coreceptors that may facilitate entry of SARS-CoV-2. Thus, although it is intriguing that CD4+ T cells may be susceptible to SARS-CoV-2, our data show that these cells are not permissive to SARS-CoV-2 replication in vitro. More studies are required to fully identify the effects of SARS-CoV-2 entry in CD4+ T lymphocytes.

In conclusion, we report that although a human lung cell line supported replication of SARS-CoV-2, the virus did not propagate in any of the tested immune cell lines or primary human immune cells. Although we did not observe a productive infection in CD4+ primary T lymphocytes, we observed virus-like particles in these cells by electron microscopy. Thus, SARS-CoV-2 can enter CD4+ primary T lymphocytes but is unable to replicate efficiently. Our data shed light on a wider range of human cells that may or may not be permissive for SARS-CoV-2 replication, and our study strongly suggests that the human immune cells tested do not support a productive infection with SARS-CoV-2.

Appendix Supplementary results from study of isolation, sequence, infectivity, and replication kinetics of severe acute respiratory syndrome coronavirus 2.
