# Supplementary material for: Isolation, Sequence, Infectivity, and Replication Kinetics of Severe Acute Respiratory Syndrome Coronavirus 2
Source: Emerg Infect Dis. 2020 Sep;26(9):2054–63. doi: 10.3201/eid2609.201495 (PMC7454076; doi:10.3201/eid2609.201495)
Supplement: Appendix — Supplementary results from study of isolation, sequence, infectivity, and replication kinetics of severe acute respiratory syndrome coronavirus 2. [file 20-1495-Techapp-s1.pdf]

Article DOI: https://doi.org/10.3201/eid2609.201495

# Isolation, Sequence, Infectivity, and Replication Kinetics of Severe Acute Respiratory Syndrome Coronavirus 2

## Appendix

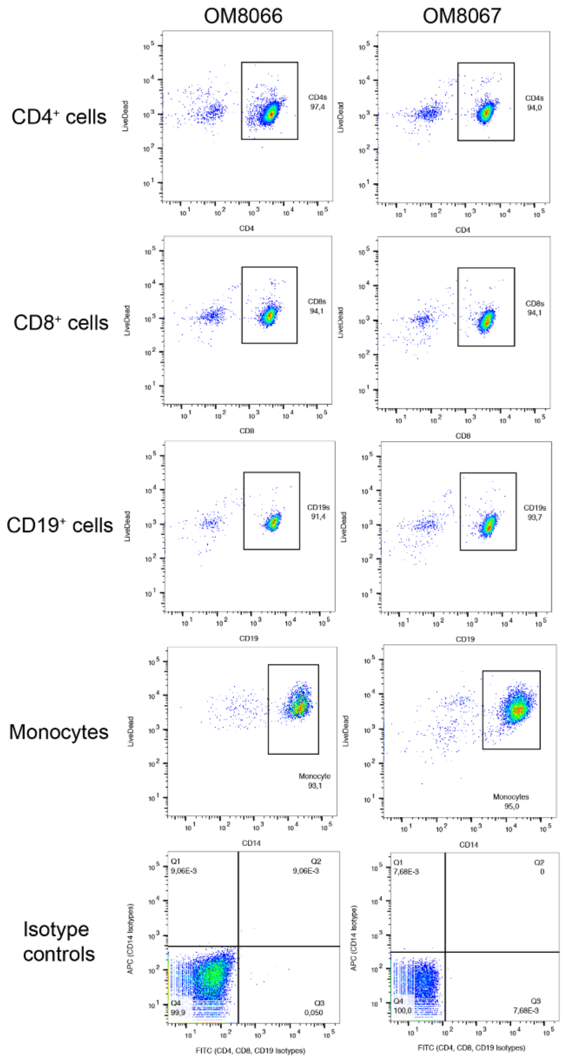


**Appendix Figure 1.** Percentage purities of cell populations that were purified from human PBMCs. Human PBMCs were collected (n = 2 independent healthy donors) and purified using cell-type specific purification kits (see Methods). Cells were stained for their respective markers and analyzed using flowcytometry. Percentage purity of each cell population are mentioned in the respective panels.

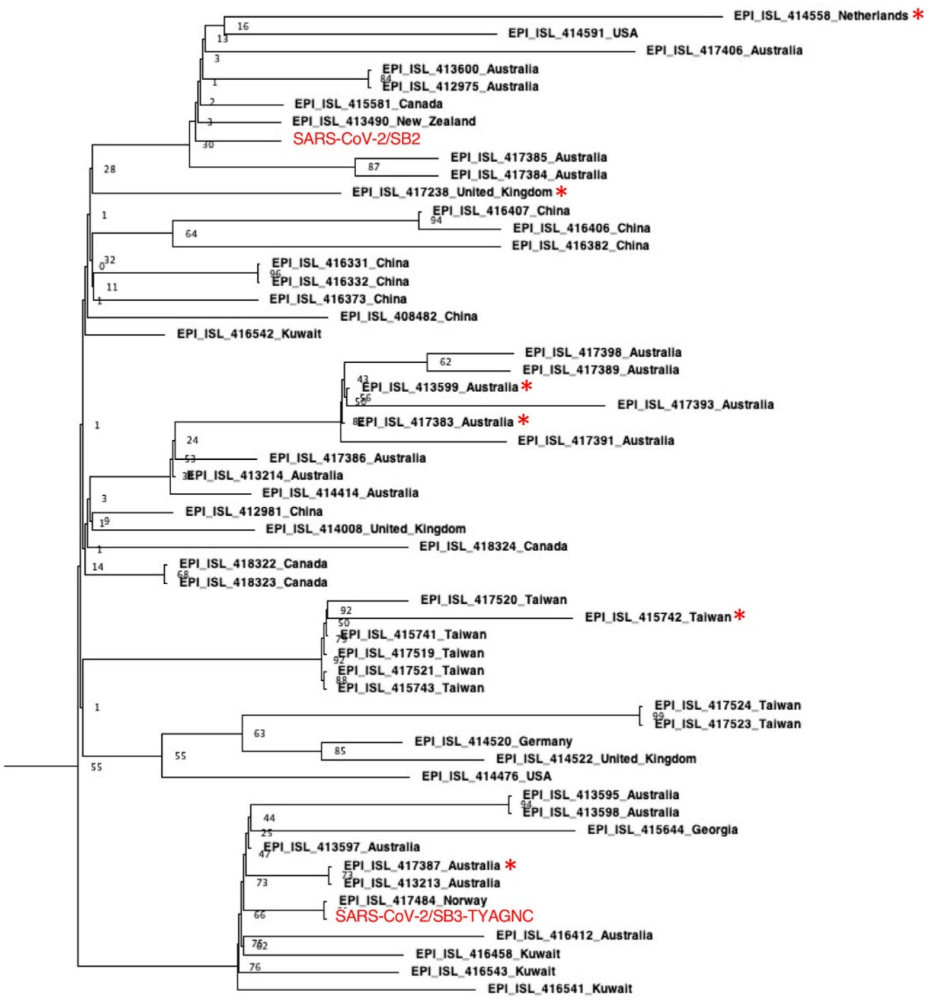


**Appendix Figure 2.** Phylogenetic relationship of SARS-CoV-2/SB2 and SARS-CoV-2/SB3-TYAGNC. Clade within a larger 1900 GSIAID isolate phylogenetic tree containing both SARS-CoV-2/SB2 and SARS-CoV-2/SB3_TYAGNC, constructed using maximum likelihood based on a multiple sequence alignment and RAxML-HPC BlackBox with GTRGAMMA + I among-site rate variation. Isolates with GSIAID metadata indicating travel history associated with the Iran outbreak are marked by a red asterisk. Branch length represent evolutionary distance, while node labels represent bootstrap support.

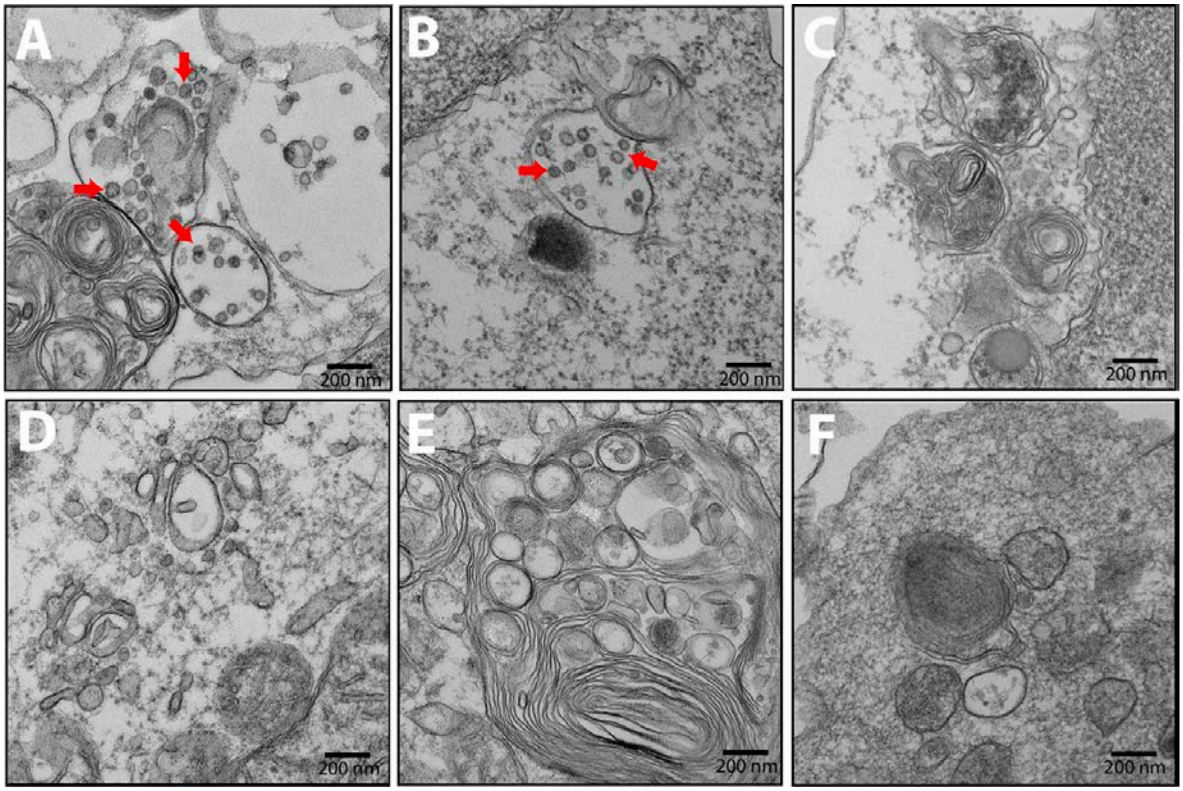


**Appendix Figure 3.** Electron micrographs of cells infected with SARS-CoV-2 for 6 hrs. To detect coronavirus-like particles in experimentally infected human structural and immune cells, we infected a range of cells with SARS-CoV-2 at a MOI of 0.01 for 6 hrs. The cells were fixed, processed and imaged using a transmission electron microscope (n = 10 fields / cell type). Representative image of each cell type is shown. Virus-like particles are indicated by red arrows. (A) Vero E6 cells. (B) $CD4^+$ PBMC. (C) $CD8^+$ PBMC. (D) $CD19^+$ PBMC. (E) Monocytes from PBMCs. (F) Other cells from PBMCs ($CD4^-$ $CD8^-$ $CD19^-$ cell populations).

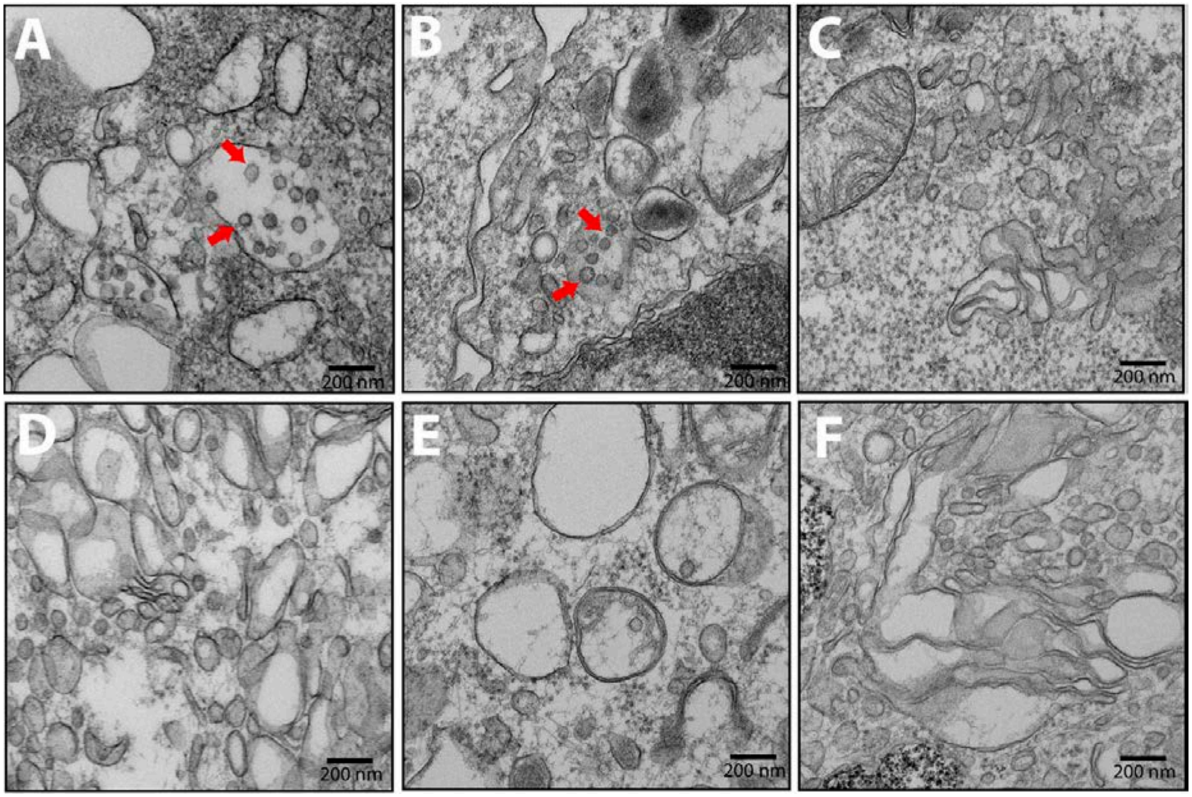


**Appendix Figure 4.** Electron micrographs of cells infected with SARS-CoV-2 for 12 hrs. To detect coronavirus-like particles in experimentally infected human structural and immune cells, we infected a range of cells with SARS-CoV-2 at a MOI of 0.01 for 12 hrs. The cells were fixed, processed and imaged using a transmission electron microscope (n = 10 fields / cell type). Representative image of each cell type is shown. Virus-like particles are indicated by red arrows. (A) Vero E6 cells. (B) $CD4^+$ PBMC. (C) $CD8^+$ PBMC. (D) $CD19^+$ PBMC. (E) Monocytes from PBMCs. (F) Other cells from PBMCs ($CD4^-$ $CD8^-$ $CD19^-$ cell populations).
